# Efficacy of switching to dolutegravir plus rilpivirine, the small-tablet regimen, in patients with dysphagia: two case reports

**DOI:** 10.1186/s40780-017-0093-8

**Published:** 2017-09-19

**Authors:** Takefumi Suzuki, Nobuko Hara, Morichika Osa, Kazuhisa Misawa, Kazuo Imai, Yuji Fujikura, Takuya Maeda, Wataru Sonehara, Akihiko Kawana

**Affiliations:** 1 Department of Pharmacy, National Defense Medical College Hospital, 3-2, Namiki, Tokorozawa-shi, Saitama, 359-8513 Japan; 2 Division of Infectious Diseases and Pulmonary Medicine, Department of Internal Medicine, National Defense Medical College, 3-2, Namiki, Tokorozawa-shi, Saitama, 359-8513 Japan; 3 Department of Pharmacy, Mishuku Hospital, 5-33-12, Kamimeguro, Meguro-ku, Tokyo, 153-0051 Japan; 4 Department of Microbiology, Saitama Medical University, 38 Morohongo, Moroyama-Machi, Iruma-Gun, Saitama, 350-0495 Japan; 5 Center for Clinical Infectious Diseases and Research, Saitama Medical University, 38 Morohongo, Moroyama-Machi, Iruma-Gun, Saitama, 350-0495 Japan

**Keywords:** Dolutegravir, Rilpivirine, Dysphagia, NRTI-sparing

## Abstract

**Background:**

The advent of well-tolerated and effective anti-retroviral drugs against human immunodeficiency virus-1 (HIV-1) infection has been a major step forward that has achieved long-term survival in recent years. The number of HIV-1 infected patients who experience difficulty in swallowing tablets is expected to increase as the HIV-infected population advances in age or develops comorbidities or treatment sequelae affecting the central nervous system.

**Case presentation:**

Here, we describe two HIV-1-infected patients who experienced progressive dysphagia leading to inability to swallow the antiretroviral tablets included in the standard regimen. Both patients had a plasma viral load < 40 copies/mL while receiving anti-retroviral therapy with the recommended combination antiretroviral therapy (cART) regimen, but the dysphagia necessitated a switch. By switching to much smaller sized combined regimen of dolutegravir (DTG) plus rilpivirine (RPV) tablets, both of our patients were able to successfully continue treatment and maintain adherence without the need for crushing tablets or preparing an oral suspension. Additionally, switching from the recommended cART regimen to DTG plus RPV successfully maintained viral suppression. At the last available follow-up (12 months after switching to DTG/RPV), HIV-1 viral load remained below the lower limit of quantification.

**Conclusions:**

An alternative therapeutic option that takes tablet size into consideration could not only contribute to improved patient adherence, but also a reduced care burden for HIV-infected patients with dysphagia. Thus, switching to the “small-tablet regimen” of DTG plus RPV has the potential to improve the survival and well-being of patients with dysphagia.

## Background

The advent of well-tolerated and effective anti-retroviral drugs against human immunodeficiency virus-1 (HIV-1) infection has been a major step forward that has achieved long-term survival in recent years [1]. Moreover, the number of HIV-1 infected patients who experience difficulty in swallowing tablets is expected to increase as the HIV-infected population advances in age or develops comorbidities or treatment sequelae affecting the central nervous system [2]. With the long lifespan conferred by combination antiretroviral therapy (cART), the patient population affected by HIV-associated dementia (HAND) or Alzheimer’s disease is also growing. The standard regimens of cART consist of two nucleoside/nucleotide reverse transcriptase inhibitors (NRTIs) plus a third drug, so it would be beneficial to reduce both the number of drugs and the tablet size for the medication to be easy to take and have good tolerability for all patients over the long term [1, 3].

The Food and Drug Administration (FDA) has previously suggested that patient adherence to medication regimens can be influenced by the size and shape of a tablet or capsule, and size specifically as the main reason for the difficulty in swallowing [4]. NRTI combination drugs (e.g., tenofovir/emtricitabine; TDF/FTC, abacavir/lamivudine; ABC/3Tc), including all recommended and alternative regimens in 2015, are >17 mm at their largest dimension and, despite being oval-shaped, can be difficult to swallow. Conversely, liquid formulations such as Kaletra® suspension are easier to administer, but caregivers would have the burden of refrigerating and measuring the liquids each day. Furthermore, Kaletra contains a high concentration of alcohol and therefore has the potential to lead to significant alcohol toxicity. Unfortunately, accepted and sufficiently safe liquid formulations are not yet licensed in Japan.

Dolutegravir (DTG) is a next-generation anti-retroviral drug, an integrase inhibitor with a long intracellular half-life that allows once daily dosing without the need for any boosting drugs [5, 6]. Its major metabolic pathway involves uridine diphosphate glucuronosyltransferase-1A1 with a minor metabolic component of cytochrome P450 isoforms, and therefore its interactions with co-medications are quite limited [7]. It is also noteworthy that its tablet size (9.1 mm), along with that of rilpivirine (RPV) which is the smallest tablet (6.4 mm) among non-NRTIs, is small enough for most patients to take orally. In recent years, several studies have focused on DTG monotherapy or DTG-based lightened regimens because of its powerful anti-viral effect, ease of medication, and excellent tolerability [8, 9]. Although the clinical effectiveness and safety of the combined regimen with DTG plus RPV is controversial and remains to be established, it has the potential to enable continued successful treatment and maintain adherence without the need for crushing or preparing an oral suspension when administering the drug to older patients or patients with dysphagia.

We describe here two cases of HIV-1-infected patients whose comorbidities involving the central nervous system (CNS) and/or aging led to difficulty swallowing the anti-retroviral tablets. Switching from the recommended cART regimen to a “small-tablet regimen” of DTG plus RPV, after patients consented to the unestablished regimen with the aim of treating their disease, successfully maintained viral suppression.

## Case presentation

### Case 1

The patient was a Japanese man with HIV-1 infection who also had multiple system atrophy (MSA), a progressive neurodegenerative disorder characterized by cerebellar ataxia, parkinsonism, and autonomic dysfunction. At age 59 years, he presented with complaints of decreased vision and myodesopsia in both eyes. The uveitis workup revealed positive serology for syphilis and HIV-1 with a CD4 cell count of 354 cells/μL and a HIV-1 viral load of 8.3 × 10^3^ copies/mL. At age 61, he was diagnosed with left pulmonary adenocarcinoma of pathological stage pT3N0M0, pStage IIB and underwent left upper lobectomy and irradiation with 60 Gy (total dose). Prior to this surgery, anti-retroviral therapy was initiated with a cART regimen of raltegravir (RAL) plus TDF/FTC according to the national protocol. Although the HIV-1 viral load became undetectable during the first 12 months of therapy, he was switched to RAL plus ABC/3Tc at age 62 because he subsequently presented with reduced renal function as a side effect of the original therapy. Thereafter, he experienced asymptomatic orthostatic hypotension, slowed movement, progressive gait instability, and severe constipation. All these symptoms and signs persisted without cognitive changes, and co-treatment with levodopa/carbidopa and a dopamine agonist was started. At a medical evaluation at age 65 years in our hospital, brain magnetic resonance imaging (MRI) showed the “hot cross bun” sign in the pons, as well as cerebellar and brainstem atrophy [10]. Finally, MSA was diagnosed and taltirelin hydrate was added to his treatment. However, symptom progression continued, and additional progressive symptoms of dysphagia became apparent; therefore, oral tablets of levodopa/carbidopa, a dopamine agonist, and taltirelin hydrate had to be provided after crushing.

**Fig. 1:**
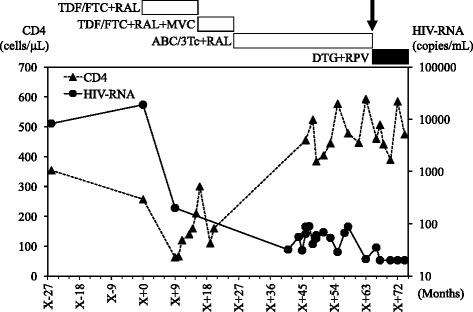
Plasma viral load and CD4 cell count follow-up from 2008 to 2016 in Case 1. Arrow indicates the time of switching to DTG/RPV dual therapy. X + 0 indicates the time of first administration of anti-retroviral drugs. Abbreviations: TDF/FTC, tenofovir/emtricitabine; ABC/3Tc, abacavir/lamivudine; RPV, rilpivirine; DRV/r, darunavir/ritonavir; MVC, maraviroc; RAL, raltegravir; DTG, dolutegravir

At age 66, he was switched to DTG/RPV dual therapy because the original anti-retroviral drug tablets were too large for him to take orally. At the last available follow-up (12 months after switching to DTG/RPV), HIV-1 viral load remained below the lower limit of quantification (< 40 copies/mL) and CD4 cell count was maintained at 474 cells/mL (Fig. 1). Although the dysphagia persisted and progressed, this change to a “small-tablet regimen” reduced the burden on his caregivers at home and enabled the patient to take the tablets on his own without needing to crush them for oral suspension.

#### Case 2

The patient was a Japanese woman with HIV-1 infection and neurologic sequelae with progressive multifocal leukoencephalopathy (PML) showing fatal subacute demyelinating disease of the brain that occurs in immunosuppressed patients. At age 30 years, she presented to our hospital with complaints of progressive confusion, severe dyspnea, and asthenia. Blood testing showed a positive serology for HIV-1 with a CD4 cell count of 116 cells/μL and a HIV-1 viral load of 7.2 × 10^3^ copies/mL. She underwent an extensive examination and based on clinical and brain MRI findings was finally diagnosed with pneumocystis pneumonia (PCP) complicated with possible PML. Despite attempts to optimize gas exchange and control infection with various antimicrobial agents, she required intubation and mechanical ventilation for a long duration. Anti-retroviral therapy was introduced via the feeding tubes primarily with crushed tablets of lopinavir/ritonavir (LPV/r) plus TDF/FTC. Consequently, HIV-1 viral load decreased and became consistently undetectable. She was also administered enteral nutrition via the same feeding tube. At age 32, she was switched to cART with EFV plus ABC/3Tc because she presented with pathological bone fractures and demineralization. Alongside intensive anti-retroviral treatment, simultaneous dysphagia and respiratory rehabilitation for more than 3 years led to progressive improvement of in her symptoms [11]. At age 36, she was successfully extubated without respiratory distress and was able to resume oral feeding. However, because the original anti-retroviral drug tablets were too large for her to take orally, she was switched to DTG/RPV dual therapy.

**Fig. 2:**
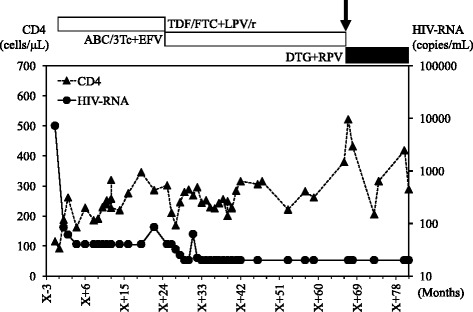
Plasma viral load and CD4 cell count follow-up from 2009 to 2016 in Case 2. Arrow indicates the time of switching to DTG/RPV dual therapy. X + 0 indicates the time of first administration of anti-retroviral drugs. Abbreviations: TDF/FTC, tenofovir/emtricitabine; ABC/3Tc, abacavir/lamivudine; RPV, rilpivirine; LPV/r, lopinavir/ritonavir; EFV, efavirenz; DTG, dolutegravir

At the last available follow-up (12 months after switching to DTG/RPV), HIV-1 viral load remained below the lower limit of quantification (< 40 copies/mL) and CD4 cell count was maintained at 289 cells/mL (Fig. 2).

## Discussion

The CNS is a major site of disease in HIV-infected patients because it serves as a reservoir for the virus as well as a site for opportunistic infections [12]. Indeed, just short of 40% of HIV-infected patients will have some neuromuscular manifestations such as dysphagia. Moreover, due to the significantly reduced mortality of these patients, the impact of the aging combined with their neuromuscular issues accelerate their need for care [13]. Previously, anti-retroviral treatment itself led to side effects such as dysphagia and various gastric symptoms including nausea, vomiting, gastroesophageal reflux, and taste aversions. However, with drastic advances in cART, recent treatment strategies have led to fewer side effects, increased efficacy, and higher virologic response in clinical practice. Therefore, adherence to cART has become one of the major independent predictors of long-term treatment success in HIV patients.

In past studies, if the diameter of the tablet exceeded 8 mm, there was a high possibility that oral administration could become difficulty for adult patients. In addition, increasing tablet or capsule size is also believed to correlate with increasing difficulty with oropharyngeal transfer [14, 15]. Therefore, treatment constituting a combination of tablets, is suitable and therefore can be reasonably defined as a “small-tablet-regimen”. By switching to the much smaller tablets of the combined regimen of DTG (9.1 mm) plus RPV (6.4 mm) tablets once a day at home, both of our patients were able to continue successful treatment and maintain adherence without the need for crushing or preparing an oral suspension. Additionally, the burden on caregivers of maintaining the oral administration of the drugs was reduced compared with administering the medications via feeding tubes, and the efficacy of the drug and the stability of the preparation were also ensured [16]. Thus, switching to the “small-tablet regimen” of DTG plus RPV has the potential to improve the survival and well-being of patients with dysphagia.

The NRTI class (e.g., ABC, TDF) remains a key component of most antiretroviral regimens used in current HIV clinical practice. However, there are clinical situations in which eliminating NRTI exposure is desirable (e.g., in patients with a high risk of cardiovascular disease [17], positive HLA-B*5701 [18, 19], chronic kidney disease [20–22], or osteoporosis [23]). As noted in the 2016 guidelines from the US Department of Health and Human Services, two NRTI-sparing regimens—darunavir (DRV)/r plus RAL and LPV/r plus 3Tc—should be considered as alternative regimens when ABC or TDF cannot be used [3].

NEAT001/ANRS143, a fully powered trial, demonstrated that DRV/r plus RAL as first-line anti-retroviral therapy was non-inferior to the standard treatment and thus presented a therapeutic option for patients with baseline CD4 cell counts > 200/μL [24]. A small single-arm study of DRV/r plus RAL, however, showed high rates of virologic failure in patients with baseline viral load > 100,000 copies/mL [25]. In the GARDEL study, the LPV/r plus 3Tc regimen was better tolerated than the LPV/r plus 2-NRTI regimen, although LPV/r is not considered a viable alternative because of its metabolic complications and pill burden [26]. Together, these data suggest that NRTI-sparing regimens have weaker efficacy than the recommended regimens. On the other hand, it has been reported that the NRTI-sparing regimen was efficacious and safe as a replacement regimen especially in patients showing long-lasting virologic suppression. A particularly attractive option currently under study is the long-acting combination of an integrase inhibitor plus a non-NRTI, the most metabolism-friendly antiretroviral drug class, for the maintenance of viral suppression [27]. The SWORD-1 and SWORD-2 sponsor-initiative clinical trials are currently ongoing to assess the antiviral activity and safety of DTG plus RPV and a current antiretroviral therapy for 48 weeks in patients with suppressed viral load [28].

With the rapid increase in the number of elderly HIV-infected patients worldwide, comorbidities associated with aging, such as dysphagia, are expected to become significant factors affecting treatment outcomes and patient quality of life. An alternative therapeutic option that takes tablet size into consideration could not only contribute to improved patient adherence, but also a reduced care burden for HIV-infected patients with dysphagia. Further studies are needed to confirm the long-term efficacy and safety of the DTG plus RPV regimen in HIV-infected patients with suppressed viral load.

## Conclusions

DTG + RPV dual therapy is effective in patients with difficulty swallowing, can be considered as alternative regimens for the maintenance of viral suppression.

## Abbreviations

cART: Combination antiretroviral therapy
DTG: Dolutegravir
NRTI: Nucleoside reverse transcriptase inhibitors
PI: Protease inhibitors
RPV: Rilpivirine
